# A Novel Technique for Recurrent Tube Exposure Repair

**DOI:** 10.1155/2020/6878025

**Published:** 2020-03-02

**Authors:** J. Minjy Kang, Yen Cheng Hsia, Ying Han

**Affiliations:** UCSF Department of Ophthalmology, USA

## Abstract

**Purpose:**

Tube exposure can lead to vision-threatening consequences and requires prompt surgical attention. Posterior repositioning of the tube to the pars plana has previously been reported as a successful technique. However, this method requires a pars plana vitrectomy (PPV). Here, we describe a novel technique of repositioning the tube into the ciliary sulcus without requiring PPV.

**Methods:**

This is a retrospective interventional case report of two patients who had undergone prior glaucoma drainage device implantation and prior tube exposure repair and developed recurrent tube exposure. Tube exposure in the subjects was repaired by repositioning the tube in the ciliary sulcus.

**Results:**

The two eyes remained exposure free postoperatively with 51- and 60-month follow-ups.

**Conclusions:**

Repositioning the tube to the ciliary sulcus may be an effective technique to avoid reexposure.

## 1. Introduction

Tube exposure after glaucoma drainage device (GDD) implantation is a postoperative complication that can occur in 1-7% of cases [1, 2]. Possible mechanisms of tube exposure include conjunctival tension over the tube, desiccation from exposure, or mechanical trauma from the eyelid. Younger age, presence of ocular inflammation prior to tube exposure, and inferior placement of the GDD are also potential risk factors [1, 3, 4]. Conflicting evidence exists for other risk factors such as race, diabetes mellitus, history of prior ocular surgery, type of patch graft, and preoperative medications [1, 5, 6]. Tube exposure may lead to endophthalmitis, and therefore, it requires prompt surgical revision [7, 8].

Several surgical techniques for tube revision have been described, most involving the reinforcement of tube coverage with various types of patch grafts (e.g., Tutoplast dura mater (Biodynamics International, Inc., Tampa, FL), sclera, pericardium, and fascia lata) [9, 10]. However, direct coverage with patch grafts may lead to recurrent tube exposure over time [10, 11]. An alternative approach to avoid such recurrence is to reposition the tube posteriorly into the pars plana (PP). This reduces the extraocular portion of the tube where it is subjected to the exposure, desiccation, and mechanical trauma from the eyelid [12–14]. However, PP placement requires concurrent pars plana vitrectomy (PPV) and may subject the patient to additional perioperative risks such as retinal tears, cataract progression, macular edema exacerbation, postoperative hypotony, or retinal detachment [15–17]. Here, we reported two cases with repeated tube exposure repaired by repositioning the tubes into the ciliary sulcus (CS) without requiring PPV with long-term success.

## 2. Methods

This is a retrospective, noncomparative, interventional case series that adhered to the tenets of the Declaration of Helsinki. All surgeries were performed by one of the authors (YH) at the University of California, San Francisco in 2013, with follow-up until October 2018. Patient charts were reviewed for relevant past medical history, past surgical history, operative notes, and postoperative course.

### 2.1. Surgical Technique

**Figure 1:**
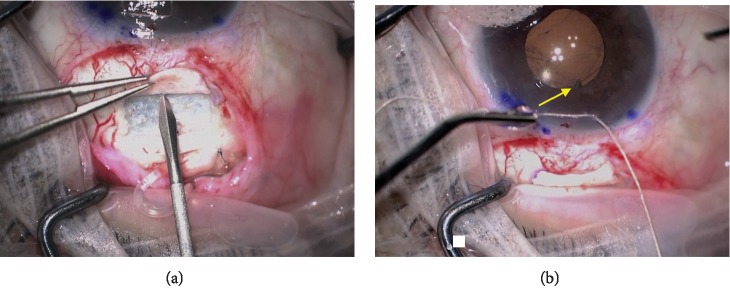
(a) A bent 20-guage MVR blade is used to enter the sulcus at 4 mm from the limbus in the plane parallel to the iris. (b) The tube (yellow arrow) placement in the CS is confirmed by direct visualization at the pupillary border.

The patient is dilated in the preoperative area. A limited conjunctival peritomy is made in the area of tube exposure. Tenon's capsule is dissected away from underlying episclera. The anterior chamber (AC) is filled with an ophthalmic viscoelastic device (OVD) via a limbal paracentesis site, and the tube is removed from the AC. The original sclerotomy site is closed with interrupted nylon sutures. OVD is injected into the CS space via the limbal paracentesis site. A bent 20-gauge MVR blade is then used to enter the CS at 4 mm from the limbus in a plane parallel to the iris (Figure 1(a)). The placement of the blade in the CS is directly visualized through the cornea, ensuring its location posterior to the iris but anterior to the intraocular lens. OVD is injected into the track using a cannula. The tube is then trimmed to a bevel-down configuration and inserted through the track. Tube placement is confirmed with direct visualization (Figure 1(b)). In cases of poor dilation, a pupil retractor can be used. A patch graft or a half-thickness scleral flap of the patient's own sclera is then used to cover the extraocular tube. Conjunctiva, or amniotic membrane in cases with inadequate conjunctiva, is secured over the patch graft.

Standard postoperative care is applied, including topical antibiotic eye drops for one week and topical steroid eye drops, such as prednisolone acetate 1%, starting at QID and tapering over four weeks.

## 3. Results

Case 1 was performed on a 60-year-old man with steroid-induced open-angle glaucoma (OAG), bilateral Baerveldt glaucoma devices, pseudophakia, and corneal edema, who initially developed tube exposure in the right eye following DSAEK/tube shortening (Table 1). Initial repair was performed by covering the tube with Tutoplast and conjunctiva. However, the tube was reexposed one-year postoperatively. The 2nd repair was performed by repositioning the tube to the CS as described above. At postoperative month 60, in the right eye, the tube remained well covered and intraocular pressure (IOP) was controlled with two drops. The patient developed endothelial graft failure in the right eye over time leading to loss of vision. Case 2 was a 75-year-old woman with Sjogren's syndrome, pseudophakia, neovascular glaucoma secondary to central retinal vein occlusion, and Ahmed glaucoma valve implantation. Tube erosion was initially repaired with a corneal patch graft and amniotic membrane graft. Recurrent tube exposure occurred 10 months later. The second repair was performed by repositioning the tube to the CS. At postoperative month 51 of the 2nd repair, the tube remained well covered and IOP was at goal on one drop. Case 2 maintained relatively stable vision at their last follow-up visit compared to their preoperative visit.

## 4. Discussion

To our knowledge, this is the first report of addressing recurrent tube exposure by repositioning the AC tube into the CS. While the number of cases is limited, the patients who have undergone tube repositioning to the CS have had excellent results with a nearly 5-year follow-up without recurrent tube exposure. This is promising given a study by Huddleston et al. reporting that 18% of the cases developed recurrent tube exposure after initial tube exposure repair (average follow-up 46.6 weeks, range 3-168 weeks) [11]. In Huddleston et al.'s study, most initial repairs used scleral patch grafts (74%) and none reported repositioning of the tube. Kalenak describes tube exposure repair with dermis graft tissue, resulting in a 13% tube reexposure rate in 30 cases (median follow-up 12 months, range 1-42 months) [10]. These reports suggest that covering the exposed tube with a patch graft may not be adequate for long-term success.

Several reasons may explain why repositioning the tube into CS prevents recurrent tube exposure. First, repositioning the tube posteriorly decreases the extraocular portion of the tube which is subject to exposure, desiccation, and repetitive mechanical trauma from the eyelid. Second, posterior positioning of the tube may help avoid a sharp angle of entry at the limbus, which can increase friction and pressure against the overlying patch graft and/or conjunctiva. Lastly, repositioning the tube posteriorly allows the tube to be covered partially by a ciliary body and by thicker tenon and conjunctiva. The combination of these factors likely contributes to the decreased rate of recurrent erosion.

These factors also likely contribute to the previously reported success in treating recurrent tube exposure by repositioning to the PP in four patients with a follow-up ranging from 2 to 42 months [13]. However, inserting a tube into the PP requires complete PPV to prevent vitreous occlusion of the tube, necessitating the added cost and time for a vitreoretinal surgeon to be present. This also increases the risk of various postoperative complications such as retinal tears or cataract progression associated with a PPV [15–17]. Our approach of inserting the tube into CS space applies a similar concept, but with a simpler approach and avoiding the additional cost and risk of complications.

Insertion of GDD tubes in the CS has been shown to be safe and effective in the past [18–21]. In fact, this technique may not only be used to treat tube exposure but may also be considered during primary implantation to avoid any future tube exposure. In a paper comparing outcomes of Ahmed GDD with tube insertion in the AC versus the CS, tube/plate exposure was reported in 2.9% in the AC group, compared to 0% in the CS group [20]. Although this difference did not reach statistical significance due to a limited sample size, it suggests a trend towards decreased exposure with posterior insertion.

In summary, we present a practical method to repair recurrent tube exposure by repositioning the tube to the CS. This method may be as effective as repositioning it to the PP, but it avoids the additional cost, operating time, and perioperative risks associated with PP tube placement.

## Figures and Tables

**Table 1:** Patient characteristics and outcomes.

Case	Glaucoma type	Time to exposure	Prior tube exposure repair	Preop VA	Postop VA	Pre IOP	Post IOP	Pre # drops	Post # drops	Follow-up
1	SI-OAG	39 mo	Conjunctiva oversewn	20/200	HM	15	11	4	2	60 mo
2	NVG	1 mo	K/AMG	HM	LP	17	19	0	1	51 mo

Abbreviations: SI-OAG=steroid-induced open angle glaucoma; NVG=neovascular glaucoma; Time to exposure=time from initial GDD implantation to first exposure of tube; K/AMG=corneal and amniotic membrane patch graft.
